# Computed tomographic pulmonary angiography and pulmonary embolism: predictive value of a d-dimer assay

**DOI:** 10.1186/1756-0500-5-104

**Published:** 2012-02-17

**Authors:** Patricia Deonarine, Carl de Wet, Alistair McGhee

**Affiliations:** 1 Radiology Department, Glasgow Royal Infirmary, 84 Castle Street, Glasgow G4 0SF, UK; 2 General practitioner and research fellow, NHS Education for Scotland, Glasgow, UK; 3 Radiology consultant, Monklands district general hospital, Lanarkshire, UK

**Keywords:** Pulmonary embolism, D-dimer, CTPA (tomography)

## Abstract

**Background:**

Computed tomographic pulmonary angiography (CTPA) is increasingly being used as first investigation for suspected pulmonary embolism (PE). The investigation has high predictive value, but is resource and time intensive and exposes patients to considerable radiation. Our aim was to assess the potential value of a negative d-dimer assay to exclude pulmonary emboli and reduce the number of performed CTPAs.

**Methods:**

All CTPAs performed in a Scottish secondary care hospital for a fourteen month period were retrospectively reviewed. Collected data included the presence or absence of PE, d-dimer results and patient demographics. PE positive CTPAs were reviewed by a specialist panel.

**Results:**

Pulmonary embolisms were reported for 66/405 (16.3%) CTPAs and d-dimer tests were performed for 216 (53%). 186/216 (86%) patients had a positive and 30 (14%) a negative d-dimer result. The panel agreed 5/66 (7.6%) false positive examinations. The d-dimer assay's negative predictive value was 93.3% (95% CI = 76.5%-98.8%) based on the original number of positive CTPAs and 100% (95% CI = 85.9%-100%) based on expert review. Significant non-PE intrapulmonary pathology was reported for 312/405 (77.0) CTPAs, including 13 new diagnoses of carcinoma.

**Conclusions:**

We found that a low d-dimer score excluded all pulmonary embolisms, after a further specialist panel review identified initial false positive reports. However, current evidence-based guidelines still recommend that clinicians combine a d-dimer result with a validated clinical risk score when selecting suitable patients for CTPA. This may result in better use of limited resources, prevent patients being exposed to unnecessary irradiation and prevent potential complications as a result of iodinated contrast.

## Background

Pulmonary embolism (PE) is associated with substantial morbidity and mortality. In the US more than 500 000 patients per year are diagnosed with pulmonary emboli, resulting in approximately 200 000 deaths [1,2]. In England and Wales there are around 65 000 cases of pulmonary emboli annually amongst hospitalized patients. The prevalence of unsuspected pulmonary embolism at post-mortem is 3-8%, figures that have changed little over three decades. The implication is that the true number of cases may be substantially higher than is currently being diagnosed [3].

The presentation, symptoms and clinical signs of pulmonary embolism varies widely between patients [4]. Clinical suspicion invariably requires further investigation to confirm or exclude PE. In Scotland, computed tomographic pulmonary angiography (CTPA) is increasingly used as the first and only investigation for this purpose [5]. This is because of greater availability and reported overall sensitivity (89-100%) of helical CT, but exposes patients to substantial ionizing radiation [6-8]. A recent review by Davies et al. found that iatrogenic radiation exposure has significant risks which are often overlooked, while it was estimated that 30% of computed tomography tests may be unnecessary [9].

D-dimer assays have low specificity, but high sensitivity and negative predictive value in most patients with suspected thromboembolism, and may be an alternative first investigation to CTPA [10]. D-dimers are degradation products of cross linked fibrin and are considered the best laboratory markers of coagulation activation [11,12]. They are commonly elevated in patients with recent surgery, malignancy or infection [3,13]. As a result the diagnostic value of d-dimer assays is higher for ambulatory patients compared with those in hospital [14].

Our main aim was to assess the potential value of a negative d-dimer assay to exclude pulmonary emboli and reduce the number of performed CTPAs. A further aim was to describe incidental but significant intrapulmonary CTPA findings that may have accounted for patients' clinical presentations.

## Results

A total of 416 CTPAs were performed during the study period. 11/416 (2.6%) CTPAs were reported as indeterminate or inconclusive and excluded from further analysis. 'Technical factors', for example inadequate contrast opacification of the pulmonary arterial tree, were stated as the reason for inconclusive studies. Of the remaining 405 CTPA studies, 226 (55.8%) were performed for female and 179 (44.2%) for male patients. The mean age of all patients was 63 years (range 20-95 and standard deviation ± 17.2).

A diagnosis of pulmonary embolism was reported for 66 (16.3%) of the remaining 405 CTPAs. The expert panel unanimously agreed that five (7.6%) of these were false positive examinations. The positive predictive value of CTPA for pulmonary embolism in this study was 92.4% (95% CI = 82.5%-97.2%).

**Figure 1:**
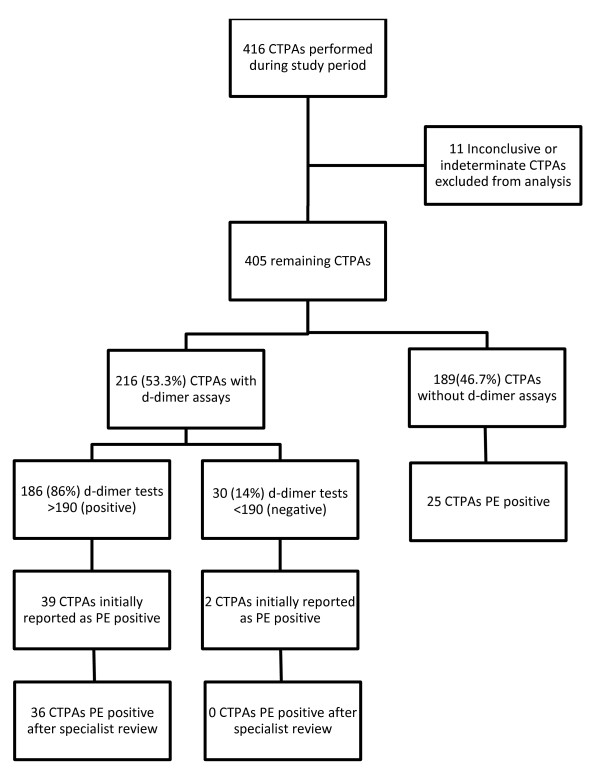
**Number of computed tomography pulmonary angiogram (CTPA) studies, d-dimer assays, and diagnoses of pulmonary embolism (PE)**.

The numbers of performed d-dimer assays, d-dimer results and CTPAs positive for pulmonary embolism are shown in Figure 1. Of the 216 (53.3%) patients who had a d-dimer assay performed, 186 (86%) had a positive and 30 (14%) a negative d-dimer result. There were initially two positive CTPAs with negative d-dimer results. Both of these were unanimously judged to be negative CTPAs (false positives) by the panel. The d-dimer assay's negative predictive value was 93.3% (95% CI = 76.5%-98.8%) based on the original number of positive CTPA reports and 100% (95% CI = 85.9%-100%) based on the panel's review. The d-dimer assay and CTPA sensitivity, specificity and predictive values are shown in Table 1. The complete data set is available as an additional file 1 (see CTPA ddimer.xls).

**Table 1:** D-dimer assay and CTPA sensitivity, specificity and predictive values (95% confidence intervals)

	D-dimer assay: Results based on number of positive CTPA results		CTPA
		
	N = 41 (initial results)	N = 36 (results after specialist review)	N = 405
Sensitivity	95.1 (82.2-99.2)	100 (88.0-100)	x

Specificity	16.0 (11.1-22.5)	16.7 (11.7-23.1)	98.5 (96.4-99.5)

Positive predictive value	21.0 (15.5-27.7)	19.4 (14.1-25.9)	92.4 (82.5-97.2)

Negative predictive value	93.3 (76.5-98.8)	100 (85.9-100)	x

The main anatomical sites of pulmonary embolism are shown in Table 2. Sub segmental embolisms were least common, being described in 4 (6%) of cases. The main findings of the CTPAs considered for analysis are shown in Table 3. Significant non-PE intrapulmonary pathology was reported for 312/405 (77.0) CTPAs. The most common reported abnormalities were pleural effusion (16.5%), bronchiectasis (10.6%) and consolidation (7.9%). There were 13 (3.2) new diagnoses of *unsuspected *carcinoma. Of the 66/405 (16.3) CTPAs reported as pulmonary embolism, 52 patients had significant additional pulmonary pathology.

**Table 2:** Classification of pulmonary emboli according to anatomical site

	No*	(%)
Large main trunk	21	(32)

Lobar artery	20	(30)

Segmental	21	(32)

Sub-segmental	4	(6)

**Total**	**66**	**(100)**

* Each PE positive case was included only once in the classification according to the largest thrombosed vessel reported

**Table 3:** The main findings of 405 CTPAs performed between 01/06/2008 and 31/07/2009 that met inclusion criteria

Main CTPA finding	No (%)*	No (%)*
No pathology		103 (25.4)

Pulmonary embolism (PE)		66 (16.3)

• PE only	14 (3.5)	

• PE with additional abnormal finding (included below)	52 (12.8)	

Significant non-PE intrapulmonary pathology		312 (77.0)

• Pleural effusion	67 (16.5)	

• Consolidation	32 (7.9)	

• Effusion *and *consolidation	27 (6.7)	

• Carcinoma	13 (3.2)	

• Lobar collapse	22 (5.4)	

• Lymphadenopathy	13 (3.2)	

• Bronchiectasis	43 (10.6)	

• Pulmonary fibrosis	7 (1.7)	

• Other (for example atelectasis)	88 (21.7)	

*Some CTPAs had more than one main finding reported. The number and percentage is the proportion of the 405 CTPAs with that specific finding

## Discussion

### Main findings

The study's main aim was to assess the potential value of a negative d-dimer assay to exclude pulmonary emboli and reduce the number of performed CTPAs. We found a low (negative) d-dimer score to have a very high negative predictive value, but that it did not exclude all pulmonary emboli based on the initial CTPA reports. However, a low d-dimer score did exclude all pulmonary emboli after a further specialist panel review identified false positive reports.

### Comparison with existing literature

Our findings are comparable to other studies in various health care settings. Dunn et al. reported a negative predictive value for d-dimer assay of 99.6% (95% CI = 98.7- > 99.9%) and suggested that negative results could help to reduce the number of performed CTPAs [15]. More recently, Eng et al. and Hirai et al. concluded that a d-dimer test alone was suitable for screening patients with a clinical suspicion of PE [16,17]. However, a number of case reports have questioned whether a negative d-dimer result alone is sufficient to exclude pulmonary embolisms [18]. There is compelling evidence that a negative d-dimer result can effectively exclude a PE when it is combined with a low pretest clinical probability score. Current best practice clinical guidance advises combining the d-dimer result with a validated tool--for example the Wells or Geneva rule--which allow risk to be quantified in a structured manner [19-22].

A further study aim was to describe incidental but significant intrapulmonary CTPA findings that may have accounted for patients' clinical presentations. Significant pathological findings were reported for the vast majority of CTPA studies. The number of CTPA reports which described previously undiagnosed malignancies was substantially higher than reported by Kino et al. [23]. These significant non-PE findings had clinical relevance for some patients and may subconsciously lead clinicians to rationalize CTPA requests. However, CTPA has certain technical limitations that reduce its potential value in assessing non-PE pathology and cannot be considered a screening tool.

The anatomical distribution of pulmonary emboli we found was comparable to that reported by Sohns et al. [24]. The majority of thrombi were diffusely distributed between the main pulmonary trunk, lobar and segmental arteries with only a small minority involving the sub-segmental vessels. All of the false positive CTPAs were initially reported as 'small, sub-segmental pulmonary embolism'. The expert panel's opinion was that in these cases small lymph nodes or veins adjacent to sub-segmental arteries had typically been misinterpreted as filling defects. It is possible that a substantial number of sub-segmental pulmonary emboli may be false positive as a result of CTPAs being interpreted in a single plane.

### Strengths and limitations

Our findings are based on a substantial sample and an additional independent panel that reviewed CTPA studies to identify false positive results. We also identified the anatomical distribution of emboli and additional intrapulmonary pathology that may have accounted for the patients' symptoms. The study has a number of limitations: CTPA findings were not linked with clinical outcomes; validated tool such as the Geneva or Wells rule was not used or recorded by clinicians requesting CTPAs; other imaging, for example doppler ultrasound, chest x-rays and ventilation perfusion scans were not considered; and more modern alternatives to the 16-slice CT scanner used in our study may have improved diagnostic accuracy.

### Implication and future research

There are clinical guidelines for health care workers investigating a patient suspected of having a PE [19-22]. It is recommended that a clinical probability assessment and d-dimer value should be combined and used to quantify the patient's risk of PE as low, moderate or high. CTPAs are only indicated for those patients judged to be at moderate or high risk. This approach is seldom used in practice, resulting in unnecessary CTPAs being performed. This is an inefficient use of limited time and resources and expose patients to avoidable irradiation and potential complications of iodinated contrast [15,18]. Further research is required to better understand the challenges in promoting and implementing the routine use of clinical risk stratification for ambulatory patients with suspected PE.

## Conclusions

A low d-dimer score had a very high negative predictive value, but did not exclude all pulmonary embolisms based on the initial CTPA reports. However, a low d-dimer score did exclude all pulmonary embolisms after a further specialist panel review identified false positive reports. A practical and evidence-based approach is to combine a d-dimer result with a validated clinical risk score to help select suitable patients for CTPA. This may result in better use of limited resources, prevent patients being exposed to unnecessary irradiation and prevent potential complications as a result of iodinated contrast.

### Availability of supporting data

The data set supporting the results of this article is included within the article and its additional file 1.

## Methods

All CTPAs that had been performed at a District General Hospital (DGH) in Lanarkshire, Scotland, in the fourteen month period from 1^st ^June 2008 to 31^st ^July 2009 were identified and retrospectively reviewed on the Hospital Information System (HIS). This sample included CTPAs requested for hospitalized and ambulatory patients. Patients were considered ambulatory if they had been referred from the accident and emergency department or from medical, surgical and oncology out-patient units. An indeterminate or inconclusive CTPA report was the only exclusion criterion.

All PE positive studies were reviewed independently by a panel consisting of three radiologists with an interest in this area who interpreted the CTPAs using axial, coronal and sagittal reformats. The initial CTPA reports were judged to be false positive only if all three panel members agreed that the study did not show sufficient evidence of PE. Data were collected for presence or absence of PE and the type of PE, whether a d-dimer assay was performed and the d-dimer result if applicable. Patients' age and gender and other reported pathological intra thoracic findings were also collected. Patient identifiers were removed and data were entered in an Excel spreadsheet. The data were exported to SPSS version 17.0 for calculation of descriptive statistics.

The d-dimer assay and CTPA sensitivity, specificity and positive and negative predictive values were calculated with 95% confidence intervals. The d-dimer assay values were calculated twice, using the initial number of positive CTPA results, and then the revised number of positive CTPA results as determined by the specialist panel. Pulmonary emboli were classified according to their anatomical distribution. Each case was included only once and grouped according to the largest thrombosed vessel reported.

### CTPA

All CTPA studies were performed using a Toshiba Aquillon 16 slice CT scanner with slice thickness set at 1 mL.

### D-dimer

Four different types of d-dimer assay formats are currently available: enzyme linked immunosorbent assay (ELISA), whole blood erythrocyte agglutination assay (SimpliRED), semiquantitative latex agglutination assays (Accuclot, Trinity Biotech, Bray) and immunochromatographic/quantitative immunoturbidimetric assays. ELISA is considered the gold standard for the determination of d-dimer concentration. It is a highly sensitive test but is time consuming and not suitable for individual patient testing. The Accuclot d-dimer assay is less sensitive, but suitable for individual patient testing [25-27]. The Trinity Amax Accuclot d-dimer assay--a semi quantitative latex agglutination assay--was used during the study period. A d-dimer value ≥190 ng/mL was considered positive (high) and < 190 ng/mL negative (low) in accordance with local guidelines and the recommendation of the Accuclot d-dimer assay manufacturer.

## Competing interests

The authors declare that they have no competing interests.

## Authors' contributions

PD helped to design the study, collected the data and helped to prepare the manuscript. CdW coded and analyzed the data and helped to prepare the manuscript. AM had the original idea for the study, was one of the 'expert' reviewers and reviewed the final manuscript. All authors read and approved the final manuscript.

## Supplementary Material

Additional file 1 **The complete data set is available as a Microsoft Excel spreadsheet and can be downloaded as an additional file (CTPA ddimer.xls)**. Click here for file
